# Promoting Stigma-Free Language in Type 2 Diabetes Care Among Primary Care Clinicians: Randomized Controlled Pilot Trial

**DOI:** 10.2196/95805

**Published:** 2026-08-11

**Authors:** Kevin L Joiner, Alexandra Agapiou, Alicia Carmichael, Brandon P Labbree, Donna Walter, Houda Tarraf, Medha Raju, Boluwatife Dogari, Nao Hagiwara, Sophia Lin, Richard Gonzalez

**Affiliations:** 1 Department of Health Behavior and Clinical Sciences, School of Nursing, University of Michigan, 426 N Ingalls St, Ann Arbor, MI, 48109, United States, 1 734-647-0127; 2 Department of Systems, Populations, and Leadership, School of Nursing, University of Michigan, Ann Arbor, MI, United States; 3 Institute for Social Research, University of Michigan, Ann Arbor, MI, United States; 4 Department of Public Health Sciences, University of Virginia, Charlottesville, VA, United States

**Keywords:** type 2 diabetes, diabetes stigma, health care communication, clinician attitudes, medical education, intervention, patient-provider interaction

## Abstract

**Background:**

The American Diabetes Association and the Association of Diabetes Care & Education Specialists recommend person-first, strengths-based, and stigma-free language in diabetes care and education. However, interventions to promote stigma-free language among primary care clinicians remain limited.

**Objective:**

This mixed methods pilot study aimed to evaluate the acceptability and preliminary effects of a diabetes stigma-reduction training module for primary care clinicians, designed to increase awareness of language used in clinician-patient interactions and explore changes in attitudes and intentions toward avoiding stigmatizing language.

**Methods:**

Twenty-one primary care clinicians completed either a stigma-reduction training module (n=12) or an active control condition (n=9). Both conditions included a standardized patient encounter, an educational video, and a guided self-reflection activity. The stigma-reduction training module included diabetes-specific content on stigmatizing language and person-first, strengths-based alternatives. Theory of Planned Behavior domain measures were administered before and immediately after the assigned condition. Attitudes and intentions were assessed precondition and postcondition. Acceptability was evaluated using a postcondition survey and semistructured qualitative interviews. Repeated-measures ANOVA was used to assess between-group changes over time, and a qualitative descriptive approach was used to explore clinicians’ perceptions.

**Results:**

Clinicians who received the stigma-reduction training module demonstrated significantly greater improvement in attitudes toward avoiding stigmatizing language compared with clinicians in the active control condition, with mean attitude scores increasing by 1.19 (SD 0.68) points versus 0.21 (SD 0.44) points, respectively. A significant condition × time interaction was observed (*F*_1,19_=14.27; *P*=.001, partial η^2^=0.43, Cohen *d*=1.67). The between-group difference in intention change was not statistically significant, with mean intention scores increasing by 0.69 (SD 1.08) points in the stigma-reduction training module condition versus 0.02 (SD 0.90) points in the active control condition (*F*_1,19_=2.33; *P*=.14, partial η^2^=0.11, Cohen *d*=0.67). Acceptability ratings were higher for the stigma-reduction training module than for the active control condition for clarity (mean 6.17, SD 0.72 vs mean 4.44, SD 1.74; *P*=.02), helpfulness (mean 6.08, SD 1.00 vs mean 4.56, SD 1.59; *P*=.03), and likelihood of recommendation (mean 6.25, SD 0.87 vs mean 4.56, SD 1.67; *P*=.02). The qualitative findings suggested that clinicians perceived the stigma-reduction training module as actionable, relevant to primary care, and useful for promoting self-reflection about habitual language use. The active control condition was generally perceived as a useful refresher but less novel and impactful.

**Conclusions:**

This pilot study suggests that a brief, theory-informed diabetes stigma-reduction training module is acceptable and associated with improved clinician attitudes toward stigmatizing language in a simulated clinical environment. Changes in intentions were descriptively in the expected direction but did not reach statistical significance. The findings should be interpreted cautiously, given the small sample size and lack of behavioral outcome assessment. Future studies should evaluate the module in larger, more diverse samples and assess whether changes in attitudes translate into sustained changes in clinician language use and patient experiences.

## Introduction

Over 38 million US adults are living with diabetes, and approximately 90% to 95% of them have type 2 diabetes (T2D) [1]. Although adults with T2D strive to lead full, productive, and healthy lives, T2D remains a complex and demanding chronic condition. Recent advances in treatments and technologies have helped prevent or delay diabetes-related complications and mortality while improving health and quality of life [2]. However, many US adults with T2D continue to face significant challenges, underscoring the need for ongoing efforts to improve diabetes care, self-management education, and support [3].

Emerging evidence suggests that adults with T2D encounter diabetes-related stigma in social, work, and health care settings [4,5]. Stigma is a social process in which an identifiable characteristic is used to label individuals or groups as distinct from a dominant reference group, typically in the context of unequal social power [6]. For people with T2D, stigma may arise from the diagnosis itself as well as from the pervasive societal judgments, assumptions, and stereotypes associated with the condition [7]. Stigma is commonly conceptualized as social stigma and self-stigma [6]. Social stigma includes perceived or experienced blame, judgment, stereotyping, rejection, exclusion, or discrimination, whereas self-stigma occurs when individuals internalize stigmatizing beliefs, leading to feelings of embarrassment or shame [6].

Among adults with T2D, diabetes-related stigma has been associated with greater diabetes distress, elevated depressive and anxiety symptoms, reduced engagement in self-care, diminished self-efficacy, poorer health care experiences, and reduced health care use [7-9]. Although research on diabetes stigma in health care settings remains limited, these findings suggest that clinician-patient communication may be an important target for stigma-reduction efforts [10].

Minimizing stigmatizing language in clinician-patient communication has therefore emerged as a priority in diabetes care [11]. According to the National Institutes of Health frameworks, clinician-patient communication is a key factor at the intersection of personal and system-level determinants [12]. Because most adults with T2D receive their diabetes care in primary care settings, effective communication between primary care clinicians and adults with T2D is essential for supporting self-management, addressing logistical and structural barriers to care, and facilitating appropriate initiation and intensification of therapies [13-16].

In 2017, the American Diabetes Association (ADA) and the American Association of Diabetes Educators, now the Association of Diabetes Care & Education Specialists (ADCES), issued a joint position statement on the use of language in diabetes care, recommending person-first, strengths-based, and stigma-free communication. This guidance is foundational to reducing diabetes stigma in clinical care [13,17]. However, the original statement appeared primarily in specialty journals, potentially limiting awareness among primary care clinicians. Primary care clinicians are therefore an important target for intervention because they play a central role in diabetes care and may be well positioned to shift clinician-patient communication away from language that contributes to diabetes stigma [15]. Nevertheless, many primary care clinicians may be insufficiently aware of the detrimental effects of diabetes stigma or the ways in which routine clinical language can perpetuate harm [11].

To address this gap, we developed a diabetes stigma-reduction training module designed to raise awareness of the ADA and ADCES language guidance and promote self-reflection on language used in primary care encounters. The module includes 3 sequential activities: a standardized patient encounter to capture language used in a simulated clinical interaction, an educational video providing guidance and examples of stigmatizing language and stigma-free alternatives, and a guided self-reflection activity in which clinicians review their recorded encounter and identify opportunities for language improvement. The module was grounded in the Theory of Planned Behavior, which posits that intentions to perform behaviors, such as avoiding stigmatizing language, are shaped by attitudes, subjective norms, and perceived behavioral control [18]. The module was also informed by growing recognition that stigma reduction efforts should look beyond a single disease or condition and address mechanisms that operate across multiple conditions [19,20]. Consistent with prior research in stigma reduction in health care, the module combined informational content with skills-building through role-play and structured, nonjudgmental reflection on personal communication behaviors [19,21].

The objective of this pilot study was to evaluate the acceptability of the diabetes stigma-reduction training module among primary care clinicians and to explore preliminary changes in attitudes and intentions toward avoiding stigmatizing language in clinician-patient interactions.

## Methods

### Study Design

This randomized controlled pilot trial was conducted and reported in accordance with the CONSORT (Consolidated Standards of Reporting Trials; Checklist 1) 2025 statement[22]. The pilot study was a randomized controlled trial using a convergent mixed methods design. Quantitative surveys and qualitative exit interviews were used to evaluate the acceptability and preliminary effects of the stigma-reduction training activities for primary care clinicians and to inform the design of a larger, multisite trial.

### Participants and Recruitment

Because all study activities were conducted in person, recruitment focused on the local geographic area. Targeted emails with informational flyers were sent to potentially eligible individuals listed in a registry of persons interested in participating in research studies maintained by the Michigan Institute for Clinical & Health Research [23]. Letters with informational flyers were also mailed to primary care clinicians practicing in the local region. In addition, flyers were posted on professional listservs and digital displays in local health systems.

Eligible participants were practicing clinicians, including physicians, nurse practitioners, and physician assistants, who provided outpatient primary care for adults with T2D on a regular basis. Eligibility was confirmed through an online screening survey.

### Procedures and Setting

All study activities took place in a simulated clinical environment at the University of Michigan HomeLab simulation facility (RRID:CR_026753), a 985-square-foot space designed to be modified into realistic living and working environments [24]. For this study, the facility was configured to mimic an outpatient clinical setting and create an immersive experience for participating primary care clinicians. Participant-facing activities occurred in a private survey and interview room, a simulated clinic hallway, and a simulated patient examination room. An adjacent audio-visual control room was used by study staff to operate recording equipment. To enhance realism, a simulated paper-based medical chart was used containing fabricated laboratory results, progress notes, and patient questionnaires.

A computer-aided lab interviewing (CALI) system, programmed within Qualtrics, was used to promote protocol standardization and maintain allocation concealment. The CALI was operated on a computer tablet by a single study team member, who was the only member of the study team to have direct contact with the participant during the in-person visit. The recruitment coordinator had contact with participants before the study, either online or by telephone. Because the randomized condition was preprogrammed by the recruitment coordinator, the research team member with direct participant contact was blinded to the participants’ randomized allocation during the study. Quantitative outcomes were collected through participant-completed electronic surveys and scored according to prespecified rules. Therefore, there was no separate human outcome assessor for the quantitative outcomes. Data analysts were not blinded during final statistical modeling because condition labels were required for the planned between-group analyses.

Potential participants completed an online screener. Once eligibility was confirmed by the recruitment coordinator, the participant consented and enrolled in the study. Following consent and enrollment, the recruitment coordinator randomly allocated the participants to the stigma-reduction training module or the active control condition using a computer-generated randomization sequence. The computer-generated randomization sequence was stored by the recruitment coordinator in a secure electronic file. Following randomization, the recruitment coordinator entered the participant’s unique ID number and their randomized condition into the CALI.

**Figure 1.**
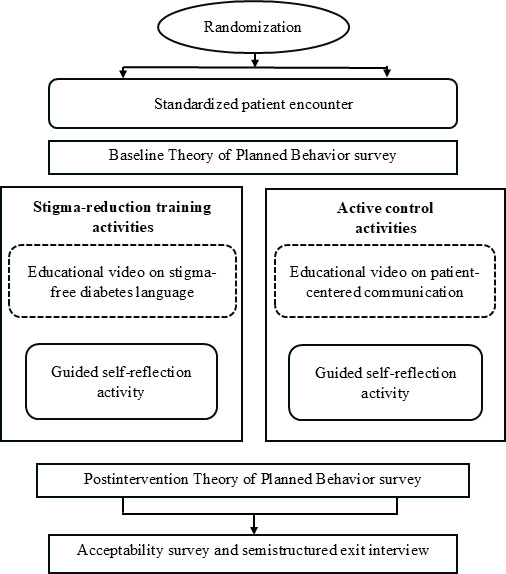
Study visit flow and randomized condition sequence.

Participating clinicians arrived in the building lobby, where they were greeted by the research staff member who implemented the study protocol and escorted to the simulation space. This research staff member was the only member of the study staff, other than the recruitment coordinator, with direct participant contact. The CALI was used by the research staff member to implement the study protocol with the participant. The CALI contained standardized scripts, reminders, and checklists to ensure consistent instructions and procedures across participants. The study visit flow is shown in Figure 1, and the full study visit protocol and choreography are provided in Multimedia Appendix 1.

### Intervention

All participants first completed a standardized patient encounter to capture baseline communication in a simulated clinical interaction. Participants then completed either the stigma-reduction training activities or the active control activities, consisting of an educational video followed by guided self-reflection using the encounter recording and transcript. The key difference between the stigma-reduction training activities and the active control activities was the educational video.

All participants completed a 15-minute role-play consultation in the simulated patient examination room with a trained actor portraying an adult with T2D attending a primary care visit for T2D with a new clinician. Two trained actors, 1 man and 1 woman, portrayed the standardized patient across study visits. Simulated paper medical charts included laboratory results, progress notes, and health questionnaires. Encounters were audio- and video-recorded and transcribed to capture natural patterns of clinician-patient communication.

Participants assigned to the stigma-reduction training module viewed a 13-minute educational video developed by members of the study team [25]. The video provided an overview of ADA and ADCES language guidance and featured contrasting vignettes illustrating stigmatizing terms and phrases alongside person-first, strengths-based alternatives. Participants assigned to the active control condition viewed a 13-minute publicly available video on general principles of patient-centered communication that did not address diabetes or stigma [26].

In both conditions, participants completed the same guided self-reflection procedure using their own encounter recording and transcript. Participants reviewed their standardized patient encounter in the private survey and interview room and identified opportunities to improve their communication based on the educational video they had viewed. Thus, the focus of reflection differed by condition because participants applied either the diabetes stigma-reduction video content or the general patient-centered communication video content. The guided self-reflection activity script and procedures are provided in Multimedia Appendix 2. In the stigma-reduction condition, this confrontation-inspired approach was intended to engage participants in recognizing their own communication patterns and forming intentions to avoid stigmatizing language in their primary care practice in the future [27-29].

The stigma-reduction training module was grounded in the Theory of Planned Behavior [18]. The educational video was designed to target attitudes by reframing stigmatizing language from routine clinical terminology to language with potential clinical harm. The guided self-reflection activity was designed to promote awareness of habitual communication patterns; in the stigma-reduction condition, this reflection was paired with educational content emphasizing stigma-free diabetes language.

### Theory of Planned Behavior Domain Measures

Preliminary effects were evaluated using surveys before and after the assigned educational video and guided self-reflection activities. The baseline survey was administered after the standardized patient encounter and before participants viewed the assigned educational video or completed the guided self-reflection. The postintervention survey was administered after the completion of the educational video and guided self-reflection. The survey was developed for the study to measure Theory of Planned Behavior domains related to stigmatizing language use in clinician-patient interactions during routine primary care for type 2 diabetes [30]. The full instrument and scoring procedures are provided in Multimedia Appendix 3. At baseline, the survey assessed 5 domains: self-reported behavior, attitudes, subjective norms, perceived behavioral control, and intentions. Immediately after exposure to the assigned educational video and guided self-reflection activity, attitudes and intentions were reassessed. These domains were prioritized for postintervention assessment because the brief study window was expected to be most sensitive to changes in attitudes and intentions, whereas subjective norms, perceived behavioral control, and self-reported behavior were not expected to change meaningfully over this period.

The Theory of Planned Behavior survey included 6 language banks: 5 representing nonrecommended or stigmatizing language and 1 representing recommended person-first, strengths-based language based on the ADA and ADCES guidance statement [17]. The 5 nonrecommended language banks included authoritative language, such as “compliant,” “non-compliant,” “compliance,” and “non-compliance”; non-person-centered language, such as “diabetic” used as a noun, for example, “Are you a diabetic?”; morally implicative language, such as “control” used as a verb, adjective, or noun, including “controlled,” “uncontrolled,” “glucose control,” and “glycemic control”; negative connotation language, such as “poor,” “poorly,” “bad,” “badly,” “fail,” and “failed”; and threatening language, such as statements focused on loss, including “You are going to end up on dialysis,” “You are going to end up blind,” or “You are going to lose a foot.” The recommended language bank included strengths-based statements focused on gain, such as “More and more people are living long and healthy lives with diabetes. Let’s work together to make a plan that you can do in your daily life.”

For each language bank, participants responded to 5 Theory of Planned Behavior domains using 7-point Likert scales. One item assessed self-reported behavior: “During the past 4 weeks, about how often have you used these words in your appointments with people with type 2 diabetes?” with anchors ranging from “all the time” to “never.” Two semantic differential items assessed attitudes: “For me, using these words in my appointments with people with type 2 diabetes is,” with anchors “good-bad” and “useful-worthless.” One item assessed subjective norms: “Most health care providers like me use these words in their appointments with people with type 2 diabetes,” with anchors “strongly agree-strongly disagree.” One item assessed perceived behavioral control: “My using or not using these words in my appointments with people with type 2 diabetes, is completely up to me,” with anchors “strongly agree-strongly disagree.” One item assessed intentions: “I intend to use these words in my appointments with people with type 2 diabetes, in the next 6 months,” with anchors “strongly agree-strongly disagree.”

Domain scores were calculated by averaging the relevant items across the 6 language banks. For the self-reported behavior, attitude, subjective norm, and intention domains, the strengths-based language bank was reverse-scored so that higher scores consistently reflected movement away from stigmatizing language and toward person-first, strengths-based language. For perceived behavioral control, all items were reverse-coded so that higher scores indicated greater perceived control over language use.

### Acceptability Measures and Qualitative Exit Interview

Acceptability was assessed using quantitative and qualitative approaches. Immediately after completing the standardized patient encounter, assigned educational video, guided self-reflection activity, and postintervention Theory of Planned Behavior survey, clinicians completed a brief 4-item acceptability survey adapted from prior work [31]. Before completing the acceptability survey, all participants were provided with a brief handout summarizing the ADA and ADCES diabetes language recommendations and were instructed to rate the study activities in relation to bringing awareness to those recommendations. Items assessed the perceived amount of information, clarity of activities, helpfulness of activities, and likelihood of recommending the activities to other clinicians. The acceptability survey is provided in Multimedia Appendix 4. All four items were rated on 7-point scales. For the amount of information item, response options ranged from 1, “too little information,” to 4, “just the right amount of information,” to 7, “too much information.” For clarity, helpfulness, and likelihood of recommendation, higher scores indicated greater acceptability of the stigma-reduction training module or the active control condition.

Semistructured exit interviews were conducted to explore clinicians’ prior exposure to ADA and ADCES language guidance, perceived barriers and facilitators to accessing and implementing these recommendations, and feedback on each activity in the stigma-reduction training module or the active control condition. The semistructured interview guide is provided in Multimedia Appendix 5. The interviews were recorded and transcribed for qualitative analysis. Demographic and clinical practice characteristics were collected online before the study visit.

### Statistical Analysis

Analyses were conducted using R (version 4.4.0; R Core Team). Analyses are presented according to condition received. Sensitivity analyses comparing as-randomized, as-treated, and per-protocol approaches yielded similar conclusions; therefore, as-treated results are reported. Means and SDs were used to describe continuous variables. Participant demographic and clinical practice characteristics were compared by condition received using Mann-Whitney *U* tests for continuous variables and Fisher exact tests for categorical variables, implemented with the *gtsummary* package.

Changes in Theory of Planned Behavior attitude and intention scores from baseline to postintervention were analyzed using 2 × 2 repeated-measures ANOVAs, with condition as the between-subjects factor and time as the within-subjects factor, implemented with the *afex* package. Planned contrasts were used to compare between-group differences at baseline and within-group pre-post changes, or simple main effects, using the *emmeans* package. Effect sizes were calculated as Cohen *d* and partial *η*^2^ using the *effectsize* package. Between-group differences in acceptability scores were compared using independent-samples *t* tests, with Cohen *d* calculated as the effect-size measure.

### Qualitative Analysis

All interviews were audio-recorded and machine-transcribed verbatim. Qualitative analysis followed a qualitative descriptive approach [32]. Two authors (AA and SL) independently reviewed and coded the transcripts, which were labeled only by participant ID. Study condition labels were withheld during coding; however, because transcripts were verbatim, analysts may have inferred condition from participants’ comments about the educational content. An initial codebook was developed through discussion with the principal investigator (KLJ) and refined iteratively. All transcripts were coded using ATLAS.ti Web (version 5.8.0). Coding discrepancies were resolved through discussion until consensus was reached. After coding was complete, codes were grouped into preliminary themes. The team refined themes through discussion, clarified theme definitions, and selected representative quotations. Years of primary care practice were aggregated into categories to protect participant confidentiality.

### Ethical Considerations

Participants who completed a study visit received an honorarium of US $80-$100, parking reimbursement, and entry into a raffle for a sports memorabilia item valued at US $250. The raffle was conducted after all participants had completed study participation, and the winner was notified by the recruitment coordinator. The protocol was deemed exempt by the University of Michigan Institutional Review Board (HUM00235534).

## Results

### Participant Flow and Characteristics

**Figure 2.**
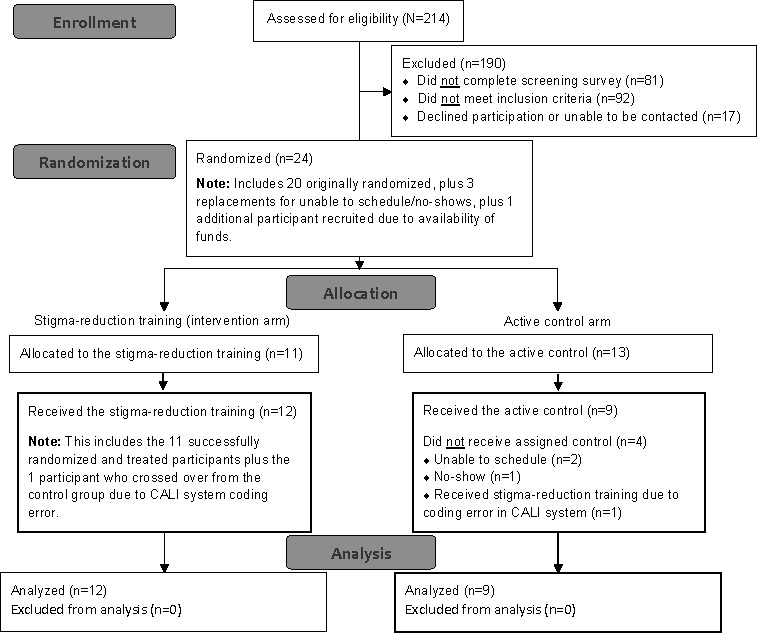
CONSORT (Consolidated Standards of Reporting Trials) flow diagram. CALI: computer-aided lab interviewing.

Clinicians were recruited and participated in the study between July 2023 and July 2024. A total of 214 individuals initiated the screening survey during this period (Figure 2). Of these, 190 were excluded: 81 did not complete the screening survey, 92 did not meet eligibility criteria, and 17 declined participation or could not be contacted. The original study design called for 20 participants randomly allocated 1:1 to the stigma-reduction training module or the active control condition. Because this was an early-stage pilot study, the sample size was selected to estimate acceptability and preliminary outcome patterns rather than to test definitive efficacy, consistent with guidance for pilot studies [33]. Before initiating a study visit, 3 enrolled participants left the study: 2 could not be scheduled, and one did not attend the scheduled visit and was subsequently lost to follow-up. Three replacement participants were recruited and randomized. In addition, sufficient remaining funds allowed the recruitment of one additional participant. Ultimately, 21 clinicians completed a study visit and were included in the analysis.

Due to a programming error in the electronic system used to implement the randomized study visit sequence, one participant randomly allocated to the active control condition received the stigma-reduction training module. Sensitivity analyses were conducted to assess the robustness of the findings to this crossover. In the as-randomized analysis, participants were analyzed according to their original randomized allocation. In the as-treated analysis, participants were analyzed according to the condition received. In the per-protocol analysis, the participant affected by the programming error was excluded. These analyses yielded consistent conclusions for attitudes. For intentions, the as-treated and per-protocol analyses were not statistically significant, whereas the as-randomized analysis reached statistical significance; therefore, intention findings were interpreted cautiously. The results of the sensitivity analyses are provided in Multimedia Appendix 6. Of the 21 clinicians who completed the study, 12 received the stigma-reduction training module, and 9 received the active control condition.

 Participant demographic and clinical practice characteristics are summarized in Table 1. The mean age of participants was 38 (SD 12) years, and most participants identified as women. Participants identified predominantly as White, followed by Asian, Middle Eastern or North African, Multiracial, and Hispanic or Latino. On average, clinicians reported 6 patients with T2D per day in clinical practice and had 7 years of clinical experience. Most were physicians, followed by nurse practitioners and physician assistants. There were no statistically significant differences between study conditions for any demographic or clinical practice characteristic.

**Table 1.** Demographic and clinical practice characteristics of primary care clinician participants by condition received.

Characteristic	Overall (n=21)	Stigma-reduction training module (n=12)	Active control condition (n=9)	*P* value^a^
Demographic characteristics				
Age (y), mean (SD)	38 (12)	40 (13)	37 (11)	.54
Gender identity, n				>.99
Man	5	3	2	
Woman	16	9	7	
Race or ethnicity, n				.43
Asian	3	3	0	
Hispanic or Latino	1	1	0	
Middle Eastern or North African	3	2	1	
Multiracial	2	1	1	
White	12	5	7	
Clinical practice characteristics				
Adults with T2D^b^ seen on a typical day, number, mean (SD)	6 (5)	7 (6)	4 (2)	.37
Practice setting, n				.62
Suburban	11	6	5	
Urban	5	2	3	
Multiple	5	4	1	
Primary care practice (y), mean (SD)	7 (8)	6 (7)	8 (9)	.94
Medical profession, n				.80
Physician	13	8	5	
Nurse practitioner	7	4	3	
Physician assistant	1	0	1	

a Mann-Whitney *U* tests were used for continuous variables, and Fisher exact tests were used for categorical variables.

b T2D: type 2 diabetes.

### Quantitative Findings

Separate 2 × 2 mixed ANOVAs were used to assess changes in attitudes and intention scores regarding stigmatizing language (Table 2). Participants exposed to the stigma-reduction training module demonstrated significantly greater improvement in attitude scores over time than participants in the active control condition, as indicated by a significant condition-by-time interaction (*F*_1,19_=14.27; *P*=.001). This represented a large effect (Cohen *d*=1.67, partial *η*^2^=0.43). For intention scores, the between-group difference in change over time was not statistically significant (*F*_1,19_=2.33; *P*=.14). However, the effect was in the expected direction, with greater improvement in the stigma-reduction training module condition. The magnitude of this effect was medium (Cohen *d*=0.67, partial *η*^2^=0.11).

Acceptability ratings were higher among participants who were exposed to the stigma-reduction training module for clarity, helpfulness, and likelihood of recommendation (all *P*<.05; Table 3). Perceptions of the amount of information conveyed did not differ significantly between groups (*P*=.37), with both groups rating the amount of information close to “just the right amount.”

**Table 2.** Theory of Planned Behavior domain scores by condition received^a^.

Domain and condition	Baseline score	*P* value^b^	Postcondition score	Change from baseline	*P* value^c^	Partial *η*^2c^(95% CI)	Cohen *d*^c^ (95% CI)
Self-reported behavior		.41			N/A^d^	N/A	N/A
Stigma-reduction module, mean (SD)	4.57 (0.64)		—^e^	N/A			
Active control, mean (SD)	4.74 (0.80)		—^e^	N/A			
Attitudes		.06			.001	0.43 (0.15-1.00)	1.67 (0.64 to 2.66)
Stigma-reduction module, mean (SD)	4.61 (0.53)		5.80 (0.68)	1.19 (0.68)			
Active control, mean (SD)	5.28 (0.80)		5.49 (0.87)	0.21 (0.44)			
Subjective norms		.75			N/A	N/A	N/A
Stigma-reduction module, mean (SD)	3.39 (0.72)		—^e^	N/A			
Active control, mean (SD)	3.46 (0.78)		—^e^	N/A			
Perceived behavioral control		.66			N/A	N/A	N/A
Stigma-reduction module, mean (SD)	6.39 (0.80)		—^e^	N/A			
Active control, mean (SD)	6.24 (0.84)		—^e^	N/A			
Intentions		.17			.14	0.11 (0.00-1.00)	0.67 (−0.22 to 1.56)
Stigma-reduction module, mean (SD)	3.69 (0.76)		4.39 (0.98)	0.69 (1.08)			
Active control, mean (SD)	4.17 (0.50)		4.19 (1.05)	0.02 (0.90)			

a Attitudes and intentions were assessed after the standardized patient encounter, before the assigned educational video and guided self-reflection activity, and again immediately after those activities. Self-reported behavior, subjective norms, and perceived behavioral control were assessed at baseline only.

b Baseline between-group comparison.

c The *P* value and partial *η*2 are from the condition × time interaction. Cohen *d* represents the between-group difference in change scores.

d Not applicable.

e Not available.

**Table 3.** Acceptability scores by condition received^a^.

Acceptability component	Stigma-reduction module (n=12), mean (SD)	Active control (n=9), mean (SD)	*P* value^b^
Amount of information	3.75 (0.62)	3.33 (1.22)	.37
Clarity of activities	6.17 (0.72)	4.44 (1.74)	.02
Helpfulness of activities	6.08 (1.00)	4.56 (1.59)	.03
Likelihood of recommending	6.25 (0.87)	4.56 (1.67)	.02

a All acceptability components were rated on 7-point scales.

b *P *values are from independent-samples *t* tests.

### Qualitative Findings

The semistructured exit interviews highlighted three main themes: (1) Integration of communication strategies in clinical practice; (2) Engagement in self-reflection and pursuit of continuous improvement; and (3) Implementation challenges, convenience, and engagement.

#### Theme 1: Integration of Communication Strategies in Clinical Practice

Several clinicians in the stigma-reduction training module arm described the module as providing education and opportunities for self-reflection to help them consider avoiding stigmatizing language and using stigma-free alternatives in clinical practice. Some commented on the clear, pragmatic approach of the stigma-reduction training module, noting that the content aligned closely with primary care realities and evolving standards. As one clinician explained:

*You want to know what you’re doing actually has an impact. Sometimes…this kind of thing feels nebulous, but this training felt actionable, like something you could really use.*

[Male, physician, primary care practice 5‐10 years]

Several described the stigma-reduction training module as reflecting a broader transformation in health care norms. One participant stated:

*It’s a paradigm shift…Terms like “compliance” are being phased out for “adherence,” and that’s really important.*

[Male, physician, primary care practice less than 5 years]

Such comments highlight how, for some participants, the intervention aligned with their interest in language choices that reflect changes in practice.

By contrast, some clinicians who received the active control condition saw the content as largely familiar and less likely to influence established routines. One participant summarized this perspective:

*The video was a reminder of the basics…. At my level, it felt like things I’d seen plenty of times…helpful for students, but not very new.*

[Physician, primary care practice less than 5 years]

This suggests that, while general communication refreshers have value, targeted, recommendation-based interventions may do more to catalyze meaningful practice change.

#### Theme 2: Engagement in Self-Reflection and Pursuit of Continuous Improvement

Some participants who received the stigma-reduction training module commented on the value of self-reflection promoted by reviewing their own recorded encounters. Several described increased awareness of their habitual language and a willingness to alter established communication patterns. One participant stated:

*Looking at my own speech, I realized how much I use words like “uncontrolled.”…I often rely on scare tactics, thinking they’ll motivate patients.…This made me see that being more positive is much more impactful.*

[Female, physician, primary care practice more than 10 years]

One participant expressed that the stigma-reduction training module fostered immediate motivation for change:

*Using neutral language…instead of negative warnings creates more productive conversations.…I can start changing that tomorrow.*

[Female, physician, primary care practice less than 5 years]

One clinician with extensive practice experience in primary care described the activities in the stigma-reduction training module as providing “more meaningful insights and opportunities for reflection” than didactic training alone (female, physician, primary care practice more than 10 years). This suggests that experiential learning, such as video and transcript review, has the potential to promote self-awareness.

Among those who received the active control condition, some participants said that even when they recognized the usefulness of improved communication, practical limitations often hampered their ability to change. One clinician expressed:

Usually, I’ll say, “How does this plan sound to you?”…But visits are so rushed…15 minutes each, back-to-back…it’s hard to be as thorough as these recommendations suggest.

[Female, physician, primary care practice less than 5 years]

Comments such as this underscore the role of systemic and contextual challenges.

#### Theme 3: Implementation Challenges, Convenience, and Engagement

Some participants reflected on both logistical and motivational factors affecting engagement in continuing education and professional development. Several clinicians who received the stigma-reduction training module commented on the value of structured opportunities to improve communication style. One stated:

*The farther you get from training, the fewer chances you have to step back and assess yourself.…You get stuck in your routines and stop thinking about your delivery.*

[Female, physician, primary care practice 5‐10 years]

Several highlighted the importance of authoritative guidance in motivating real change:

*Having the actual guidelines helped.…With just opinions, I’m skeptical, but guidelines make me more willing to change my language.*

[Female, physician, primary care practice less than 5 years]

Conversely, one participant who received the control condition questioned the added benefit of the general communication training module amid competing priorities.

*Honestly, at this stage, it’s mostly reinforcing things I already do.…It might work for students, but for most of us the issue really comes down to lack of time.*

[Male, physician, primary care practice less than 5 years]

Even so, one of the clinicians who received the control condition agreed that reviewing their own standardized patient encounter was valuable.

*Seeing my body language and eye contact from the patient’s point of view…honestly, that was the most helpful part.*

[Female, nurse practitioner, primary care practice 5‐10 years]

## Discussion

### Principal Findings

This pilot study suggests that a brief, theory-informed stigma-reduction training module is acceptable and may improve primary care clinicians’ attitudes toward avoiding stigmatizing language in T2D care. Compared with the active control condition, the stigma-reduction training module was rated significantly higher in clarity, helpfulness, and likelihood of recommendation. The survey findings showed a significant improvement in attitudes toward avoiding stigmatizing language among clinicians who received the stigma-reduction training module. Changes in intentions were descriptively in the expected direction but did not reach statistical significance.

The primary quantitative finding, a significantly greater improvement in attitudes in the stigma-reduction training module condition compared with the active control condition, aligns with the Theory of Planned Behavior. Within this framework, attitudes are a key antecedent of behavioral intention. The observed improvement suggests that the module may have shifted how clinicians appraise stigmatizing language, from routine or familiar clinical terminology to language with potential to cause harm. Although the between-group change in intentions did not reach statistical significance, the descriptive pattern was in the expected direction. This finding should be interpreted cautiously and does not provide evidence that the module changed clinicians’ intentions.

The qualitative findings helped to explain these quantitative results. Clinicians who received the stigma-reduction training module described the training as practical, relevant, and immediately applicable to clinical care. They emphasized the value of concrete language alternatives and noted that the module aligned with evolving professional standards for person-centered diabetes communication. Several participants described the guided self-reflection activity as especially useful because reviewing their own recorded standardized patient encounters allowed them to recognize habitual language patterns that may otherwise have gone unnoticed. For example, one clinician described becoming aware of relying on negative warnings and expressed readiness to begin using more neutral language immediately. These findings suggest that the combination of diabetes-specific language guidance, simulation, and structured reflection may be particularly useful for promoting awareness and motivation for communication change.

This study also highlights the added value of a targeted, guideline-linked intervention compared with general communication training. Participants in the active control condition often described the general patient-centered communication video as familiar or more appropriate for learners earlier in training. In contrast, participants who received the stigma-reduction training module described the content as actionable and connected to authoritative recommendations. This distinction suggests that primary care clinicians may be more engaged by communication training when it is specific, evidence-based, and directly relevant to a clinical challenge they encounter in practice.

To our knowledge, this is one of the first educational modules to combine a standardized patient encounter, video self-review, transcript-guided reflection, and theory-informed content to target stigma reduction in clinician-patient communication about T2D. This multimodal design allowed clinicians to observe and reflect on their language in a realistic context while receiving concrete strategies for adopting person-first, strengths-based alternatives. Prior research using simulated encounters and video-based reflection supports the value of self-review as a nonthreatening approach for helping learners identify opportunities for improvement [34]. This was particularly relevant to the present module, in which standardized patients did not provide direct feedback, preserving the authenticity of the encounter and allowing clinicians to identify their own opportunities for language change.

Participants also identified implementation challenges, including time constraints and the need for practical language tools. These findings underscore the importance of developing ready-to-use resources that can be integrated into routine primary care workflows. Because both study conditions were similar in duration, the greater emphasis on time constraints among active control participants may reflect differences in perceived relevance or motivation rather than time burden alone. Nevertheless, time pressure is a real and persistent feature of primary care, and future implementation efforts should address both clinician engagement and workflow feasibility.

Finally, the findings are consistent with habit-breaking approaches to behavior change, which emphasize awareness, motivation, concrete alternative strategies, and continued practice [35]. The stigma-reduction training module appeared to address several of these elements by increasing awareness of language use, motivating clinicians to reconsider familiar terminology, and providing actionable alternatives. However, the single-session format limited opportunities for repeated practice and reinforcement [36]. Future studies should examine whether booster sessions, reminders, longitudinal reflection, or integration into continuing medical education can support sustained changes in clinician language use in real-world practice.

### Limitations and Future Directions

This pilot was limited by its small sample size and potential self-selection bias, as clinicians with a greater interest in communication quality may have been more likely to participate. The simulated clinical environment may not fully capture the complexity of real-world primary care practice, and the brief follow-up window precluded evaluation of changes in self-reported clinician behavior. In addition, the study did not assess postintervention changes in perceived behavioral control. Future studies should assess whether changes in clinicians’ attitudes and intentions are sustained over time and whether they translate into changes in clinician language use and patient experiences.

The small sample size also limits precision in estimating effect sizes. Although baseline attitude scores did not differ statistically between groups, the stigma-reduction training module group had lower baseline attitude scores than the active control condition, creating greater room for improvement and raising the possibility that regression to the mean contributed to this observed change. Consistent with the stage model of behavioral therapies research, this study should therefore be interpreted as an early-stage pilot trial intended to evaluate acceptability, feasibility, and preliminary outcome patterns rather than definitive efficacy [33]. The large observed effect on attitudes should be interpreted with caution, but it provides an empirical signal supporting a more definitive, powered randomized trial.

Recruitment was protracted, spanning over 12 months to enroll 24 clinicians. This raises feasibility considerations for future trials and is consistent with prior literature documenting challenges in recruiting community-based primary care clinicians [37]. The high participation burden of this pilot, which required an in-person, recorded standardized patient encounter, likely contributed to the extended recruitment timeline [38]. Future studies should consider more flexible recruitment and participation strategies, including remote or hybrid formats.

Although qualitative analysts were not provided with study condition labels, full blinding was not possible because verbatim exit interview transcripts may have included comments that revealed the condition received.

### Conclusions

In summary, this mixed methods pilot study suggests that a brief, multicomponent, theory-informed stigma-reduction training module is feasible, acceptable, and associated with improved primary care clinicians’ attitudes toward avoiding stigmatizing language in T2D care. Changes in intentions were descriptively in the expected direction but did not reach statistical significance. Given its strong acceptability, this module may hold promise for integration into continuing medical education and professional development. Future studies should assess long-term effects, feasibility in real-world clinical settings, changes in clinician language use, and impact on patient experiences and outcomes, with the goal of advancing more compassionate and equitable diabetes care.

## Supplementary material

10.2196/95805 Multimedia Appendix 1 Study visit protocol and choreography.

10.2196/95805 Multimedia Appendix 2 Guided self-reflection activity.

10.2196/95805 Multimedia Appendix 3 Theory of planned behavior survey.

10.2196/95805 Multimedia Appendix 4 Acceptability survey.

10.2196/95805 Multimedia Appendix 5 Semistructured exit interview.

10.2196/95805 Multimedia Appendix 6 Sensitivity analyses by analytic approach.

10.2196/95805 Checklist 1 CONSORT checklist.

## Abbreviations

ADA: American Diabetes Association
ADCES: Association of Diabetes Care & Education Specialists
CALI: computer-aided lab interviewing
CONSORT: Consolidated Standards of Reporting Trials
T2D: type 2 diabetes
