# Supplementary material for: Promoting Stigma-Free Language in Type 2 Diabetes Care Among Primary Care Clinicians: Randomized Controlled Pilot Trial
Source: JMIR Diabetes. 2026 Aug 11;11:e95805. doi: 10.2196/95805 (PMC13460796; doi:10.2196/95805)
Supplement: Supplementary file 1 [file diabetes-v11-e95805-s001.pdf]

Multimedia Appendix 1. Study Visit Protocol and Choreography

This appendix provides the operational protocol for the in-person study visit. The purpose of this document is to describe the choreography of study personnel, participants, spaces, and technologies used to deliver the study activities in a standardized manner while maintaining blinding of participant condition assignment from study staff with direct participant contact.

A.1 Overview of Study Visit Flow

Each study visit followed the same overall sequence:

1. Arrival, greeting, and wayfinding
2. Consent review
3. Standardized patient encounter
4. Pre-intervention Theory of Planned Behavior survey
5. Educational video
6. Guided self-reflection activity
7. Post-intervention Theory of Planned Behavior survey
8. Acceptability survey
9. Semi-structured exit interview
10. Debrief, payment, and dismissal

The full visit was conducted in designated spaces within the University of Michigan HomeLab simulation research facility. The protocol was guided by a Computer-Aided Lab Interviewing guide, referred to hereafter as the CALI.

A.2 Roles and Responsibilities

Participant

The participant was a primary care clinician enrolled in the study. The participant completed the simulated clinical encounter, surveys, educational video, self-reflection activity, and semi-structured exit interview.

Interviewer

The interviewer was a trained study team member responsible for direct participant interaction. The interviewer greeted the participant, guided the participant between study spaces, read standardized instructions from the CALI, administered the study workflow, and conducted the semi-structured exit interview. The interviewer remained blinded to participant condition assignment.

Technician

The technician was a trained study team member responsible for operating the audio-visual recording system and preparing study technology during the visit. The technician did not directly interact with the participant during the study visit.

Standardized Patient

The standardized patient was a trained actor portraying an adult with type 2 diabetes in a simulated primary care encounter. The standardized patient followed a standardized role and did not provide feedback to the participant.

A.3 Study Spaces

The visit used four primary spaces:

Lobby

The lobby served as the initial arrival and dismissal location.

Survey/Interview Room

The Survey/Interview Room was a private room used for consent review, survey completion, video viewing, self-reflection, the acceptability survey, the semi-structured interview, and payment documentation.

Simulated Clinic Exam Room

The Simulated Clinic Exam Room was used for the standardized patient encounter. It contained the standardized patient, simulated clinical materials, and discrete audio-visual recording equipment.

Audio-Visual Control Room

The Audio-Visual Control Room was adjacent to the Simulated Clinic Exam Room. The technician used this room to operate cameras, microphones, recording equipment, and related technology during the standardized patient encounter.

A.4 Study Technologies and Materials

CALI Tablet

A tablet computer housed the CALI protocol. The CALI displayed standardized scripts, interviewer prompts, checklists, survey instruments, lock screens, and participant-facing materials. At designated points, the tablet was handed to the participant for self-administered study activities. During the semi-structured interview, the tablet was also used to operate audio recording software.

Laptop

A laptop was used during the guided self-reflection activity. The laptop was pre-loaded with the participant's recorded standardized patient encounter.

Audio-Visual Control Panel

The control panel in the Audio-Visual Control Room was used by the technician to operate the cameras and microphones in the Simulated Clinic Exam Room.

Simulated Paper Chart

A simulated paper medical chart was placed outside the Simulated Clinic Exam Room. It contained fabricated medical information relevant to the standardized patient encounter, including encounter instructions and simulated clinical records.

Printed Transcript

A transcript of the participant's standardized patient encounter was prepared for use during the guided self-reflection activity.

Laminated Activity Instructions

Laminated instruction sheets were used to support participant completion of activities when the interviewer was not present in the room.

Diabetes Language Recommendation Handout

A handout summarizing diabetes language recommendations from the American Diabetes Association and the Association of Diabetes Care & Education Specialists [1] was provided before the acceptability survey and referenced during the semi-structured exit interview.

A.5 CALI Procedures for Standardization and Blinding

The CALI was used to standardize the visit sequence, interviewer wording, participant-facing instructions, and staff checklists. The interviewer followed the CALI throughout the visit.

The CALI included several features designed to maintain study fidelity and blinding:

Standardized Scripts

The CALI displayed language for the interviewer to read aloud to the participant at specific points in the visit.

Staff-Only Prompts

The CALI also displayed reminders, checklists, and operational instructions intended only for study staff. These prompts were not read aloud to the participant.

Participant-Facing Sections

At designated points, the interviewer handed the tablet to the participant. The participant then completed surveys or viewed assigned materials independently.

Gates

A gate occurred when the CALI instructed the interviewer to stop, hand the tablet to another user, leave the room, or wait for a specific task to be completed before continuing.

Lock Screens

Lock screens prevented the participant from advancing into staff-only or subsequent sections of the protocol. When the participant reached a lock screen, the interviewer resumed possession of the tablet and entered a passcode to continue.

Automated Condition Assignment

The participant's educational video assignment was loaded into the CALI based on the participant's study ID and a secure study panel. The CALI displayed the assigned video to the participant without revealing the condition assignment to the interviewer.

Together, these CALI features supported standardized administration of the protocol and maintained interviewer blinding to participant condition.

A.6 Detailed Visit Protocol

Step 1. Arrival, Greeting, and Wayfinding

Location: Lobby
Individuals involved: Participant, Interviewer
Technology/materials: CALI tablet; lobby check-in system, if applicable

Procedure

1. The participant arrived at the study site lobby.
2. If the participant arrived before the scheduled appointment time, they used the automated lobby check-in system, which notified the interviewer of their arrival.
3. The interviewer met the participant in the lobby.
4. Using the CALI, the interviewer confirmed:

- participant identity;
- appointment time; and
- participant availability for the full visit duration.

5. The interviewer escorted the participant from the lobby to the Survey/Interview Room.

Step 2. Consent Review

Location: Survey/Interview Room
Individuals involved: Participant, Interviewer
Technology/materials: CALI tablet; consent form, if needed for reference

Procedure

1. The interviewer seated the participant in the Survey/Interview Room.
2. The interviewer reviewed the consent information with the participant using the CALI-guided script.
3. The interviewer confirmed that the participant had previously reviewed and electronically signed the study consent form.
4. The interviewer answered any procedural questions before continuing.

Step 3. Standardized Patient Encounter

Location: Hallway outside Simulated Clinic Exam Room; Simulated Clinic Exam Room; Audio-Visual Control Room
Individuals involved: Participant, Interviewer, Standardized Patient, Technician
Technology/materials: CALI tablet; simulated paper chart; laminated encounter instructions; audio-visual recording system; control panel

The standardized patient encounter took place in the Simulated Clinic Exam Room. The participant completed the encounter with a trained standardized patient actor while the technician monitored and recorded the encounter from the adjacent Audio-Visual Control Room.

Procedure

1. The interviewer escorted the participant from the Survey/Interview Room to the hallway outside the Simulated Clinic Exam Room.
2. The simulated paper chart and written encounter instructions were available in a wall-mounted holder outside the exam room.
3. Using the CALI, the interviewer read standardized instructions to the participant. These instructions directed the participant to:
   - review the simulated medical chart;
   - read the written encounter instructions; and
   - enter the Simulated Clinic Exam Room when ready.

4. The interviewer then left the hallway area and moved to a separate location where the standardized patient encounter could not be overheard.
5. The participant reviewed the written materials and entered the Simulated Clinic Exam Room independently.
6. Entry into the room initiated the timed encounter period.
7. The participant completed the standardized patient encounter with the standardized patient.
8. The technician monitored and recorded the encounter from the Audio-Visual Control Room using the control panel.
9. The encounter ended in one of two ways:
   - after 15 minutes had elapsed, the interviewer returned and knocked on the exam room door to end the encounter; or
   - before 15 minutes, if the participant determined that the encounter was complete and exited the room.
10. After the encounter ended, the interviewer escorted the participant back to the Survey/Interview Room.
11. The technician saved the audio-video recording and initiated preparation of materials needed for the later self-reflection activity.

Step 4. Pre-Intervention Theory of Planned Behavior Survey

Location: Survey/Interview Room
Individuals involved: Participant, Interviewer
Technology/materials: CALI tablet

Procedure

1. The interviewer read the CALI-scripted instructions for the pre-intervention survey.
2. The interviewer handed the CALI tablet to the participant.
3. The participant self-administered the pre-intervention Theory of Planned Behavior survey on the tablet.
4. After completing the survey, the participant reached a CALI lock screen.
5. The participant returned the tablet to the interviewer.
6. The interviewer entered the passcode to advance the CALI to the next section.

Step 5. Educational Video

Location: Survey/Interview Room; hallway outside Survey/Interview Room
Individuals involved: Participant, Interviewer, Technician
Technology/materials: CALI tablet; assigned educational video; laptop; transcript; laminated self-reflection instructions

Procedure

1. The interviewer read the CALI-scripted instructions for the educational video.

2. The interviewer placed the CALI tablet in front of the participant.
3. The interviewer instructed the participant to advance to the next screen only after the interviewer had left the room.
4. The interviewer left the Survey/Interview Room.
5. The participant advanced the CALI to the video screen.
6. The CALI displayed the participant's assigned educational video based on the study ID and condition assignment.
7. The participant viewed the educational video independently.
8. While the participant viewed the video, the technician prepared the materials for the guided self-reflection activity, including:
    - the laptop containing the participant's standardized patient encounter recording;
    - the printed transcript; and
    - laminated self-reflection instructions.
9. The technician delivered these materials to the interviewer outside the Survey/Interview Room without interacting with the participant.
10. After the video ended, the participant advanced the CALI to a lock screen.
11. The lock screen instructed the participant to open the door and notify the interviewer.
12. The interviewer re-entered the room with the self-reflection materials.
13. The interviewer entered the passcode to advance the CALI to the next section.

Step 6. Guided Self-Reflection Activity

Location: Survey/Interview Room
Individuals involved: Participant, Interviewer
Technology/materials: CALI tablet; laptop; encounter recording; printed transcript; writing tools; laminated self-reflection instructions; timer

Procedure

1. The interviewer read the CALI-scripted instructions for the guided self-reflection activity.
2. The interviewer provided the participant with:
    - the laptop containing the recorded standardized patient encounter;
    - the printed transcript;
    - writing tools for annotating the transcript; and
    - laminated activity instructions.
3. The interviewer confirmed that the participant understood the task.
4. The interviewer left the Survey/Interview Room with the CALI tablet.
5. The interviewer started a 15-minute timer.
6. The participant completed the self-reflection activity independently by reviewing the encounter recording and transcript.
7. After 15 minutes elapsed, the interviewer re-entered the room.
8. If needed, the interviewer stopped the video playback.

9. The interviewer collected or secured the study materials according to study procedures.
10. The interviewer advanced the CALI to the next section.

Step 7. Post-Intervention Theory of Planned Behavior Survey

Location: Survey/Interview Room
Individuals involved: Participant, Interviewer
Technology/materials: CALI tablet

Procedure

1. The interviewer read the CALI-scripted instructions for the post-intervention survey.
2. The interviewer handed the CALI tablet to the participant.
3. The participant self-administered the post-intervention Theory of Planned Behavior survey.
4. After completing the survey, the participant reached a CALI lock screen.
5. The participant returned the tablet to the interviewer.
6. The interviewer entered the passcode to advance the CALI to the next section.

Step 8. Acceptability Survey

Location: Survey/Interview Room
Individuals involved: Participant, Interviewer
Technology/materials: CALI tablet; Diabetes Language Recommendation handout

Procedure

1. The interviewer read the CALI-scripted instructions for the acceptability survey.
2. The interviewer provided the participant with the Diabetes Language Recommendation handout.
3. The interviewer explained that the handout would be referenced during the survey.
4. The interviewer handed the CALI tablet to the participant.
5. The participant self-administered the acceptability survey.
6. After completing the survey, the participant reached a CALI lock screen.
7. The participant returned the tablet to the interviewer.
8. The interviewer entered the passcode to advance the CALI to the next section.

Step 9. Semi-Structured Exit Interview

Location: Survey/Interview Room
Individuals involved: Participant, Interviewer
Technology/materials: CALI tablet; audio recording software; Diabetes Language Recommendation handout

Procedure

1. The interviewer read the CALI-scripted instructions for the semi-structured interview.
2. The interviewer re-oriented the participant to the Diabetes Language Recommendation handout.
3. The interviewer started the audio recording software on the tablet.
4. The interviewer conducted the semi-structured interview using the interview guide displayed in the CALI.
5. The interviewer followed the CALI guide while allowing appropriate probing and clarification consistent with semi-structured interviewing.
6. At the conclusion of the interview, the interviewer stopped the audio recording.
7. The interviewer confirmed that the recording had been saved according to study procedures.
8. The interviewer advanced the CALI to the final section of the protocol.

Step 10. Debrief, Payment, and Dismissal

Location: Survey/Interview Room; Lobby
Individuals involved: Participant, Interviewer
Technology/materials: Debrief form; honorarium materials; parking reimbursement materials; receipt documentation

Procedure

1. The interviewer provided the participant with the study debrief form.
2. The interviewer provided the participant with the study honorarium and parking reimbursement, as applicable.
3. The interviewer completed required payment and receipt documentation.
4. The interviewer answered any remaining procedural questions.
5. The interviewer escorted the participant from the Survey/Interview Room back to the lobby.
6. The participant was dismissed from the study visit.

   Note: Participants who completed a study visit were also entered into a raffle for a sports memorabilia item valued at $250. The raffle was conducted after all participants had completed study participation, and the winner was notified separately by the recruitment coordinator. The raffle was not conducted during the in-person study visit.

A.7 Choreography to Maintain Blinding

Several procedural features were used to preserve interviewer blinding to participant condition assignment:

1. **Condition assignment was embedded in the CALI workflow.**
   The interviewer entered the participant study ID, and the CALI loaded the appropriate educational video without displaying the condition assignment to the interviewer.
2. **The interviewer left the room before video assignment was displayed.**
   The participant advanced the CALI to the video screen only after the interviewer exited the room.
3. **Lock screens prevented unintended advancement.**
   After participant-facing sections, the CALI stopped at lock screens that required interviewer passcode entry before the protocol could continue.
4. **The technician prepared materials without participant interaction.**
   During the educational video, the technician prepared and delivered the laptop and self-reflection materials to the interviewer outside the Survey/Interview Room.
5. **The standardized patient encounter was not observed by the interviewer.**
   The interviewer left the encounter area before the participant entered the Simulated Clinic Exam Room and remained outside hearing range for the duration of the encounter.
6. **Participant-facing and staff-facing CALI content were separated.**
   Staff instructions, checklists, and passcode-protected sections were not visible to the participant unless specifically intended.

A.8 Choreography Summary Table

Study step	Location	Participant	Interviewer	Technician	Standardized patient	Primary technology/ materials
Arrival and wayfinding	Lobby	Arrives and checks in	Greets and escorts participant	Not involved	Not involved	CALI tablet; check-in system
Consent review	Survey/ Interview Room	Reviews consent information	Reviews consent using script	Not involved	Not involved	CALI tablet
Standardized patient encounter	Simulated Clinic Exam Room	Conducts simulated visit	Provides instructions, then leaves area	Records encounter	Portrays patient	Simulated chart; AV system
Pre-intervention survey	Survey/ Interview Room	Completes survey	Provides instructions; manages lock screen	Not involved	Not involved	CALI tablet
Educational video	Survey/ Interview Room	Watches assigned video	Leaves room; remains blinded	Prepares self-reflection materials	Not involved	CALI tablet; assigned video
Guided self-reflection	Survey/ Interview Room	Reviews recording and transcript	Provides materials, leaves, times activity	Not directly involved	Not involved	Laptop; transcript; instructions

Post-intervention survey	Survey/ Interview Room	Completes survey	Provides instructions; manages lock screen	Not involved	Not involved	CALI tablet
Acceptability survey	Survey/ Interview Room	Completes survey	Provides handout and instructions	Not involved	Not involved	CALI tablet; handout
Semi-structured interview	Survey/ Interview Room	Responds to interview questions	Conducts and records interview	Not involved	Not involved	CALI tablet; audio recorder
Debrief and dismissal	Survey/ Interview Room; Lobby	Receives payment and debrief	Completes documentation and escorts participant	Not involved	Not involved	Debrief form; payment materials

References

[1] Dickinson JK, Guzman SJ, Maryniuk MD, O'Brian CA, Kadohiro JK, Jackson RA, D'Hondt N, Montgomery B, Close KL, Funnell MM. The Use of Language in Diabetes Care and Education. Diabetes Care. 2017 Dec;40(12):1790-1799. doi: 10.2337/dci17-0041
