# Supplementary material for: Promoting Stigma-Free Language in Type 2 Diabetes Care Among Primary Care Clinicians: Randomized Controlled Pilot Trial
Source: JMIR Diabetes. 2026 Aug 11;11:e95805. doi: 10.2196/95805 (PMC13460796; doi:10.2196/95805)
Supplement: Supplementary file 2 [file diabetes-v11-e95805-s002.pdf]

Multimedia Appendix 2. Guided Self-Reflection Activity

The guided self-reflection activity was completed after the educational video. During this 15-minute activity, participants reviewed the recording and transcript of their standardized patient encounter and reflected on their communication in light of the educational video they had just viewed. The same self-reflection instructions and procedures were used in both study conditions. The content focus of the reflection differed insofar as participants applied the content of the assigned educational video: the diabetes stigma-reduction video or the general patient-centered communication video. The annotations made during this activity were used to support participant reflection and were not scored as a study outcome.

The activity was completed privately in the Survey/Interview Room. The interviewer provided standardized verbal instructions, set up the materials, left the room during the activity, and returned when 15 minutes had elapsed.

Materials

The following materials were used for the guided self-reflection activity:

- Laptop computer pre-loaded with the participant's standardized patient encounter recording
- Printed transcript of the standardized patient encounter
- Writing instruments, including pen, pencil, and/or highlighter
- Written activity instructions

Interviewer Instructions and Participant Script

Staff-only directions are shown in *italicized brackets* and were not read aloud to participants. All other text was read aloud by the interviewer.

> *Bring the laptop, printed transcript, writing instruments, and written instruction sheet into the Survey/Interview Room.*
>
> Thank you for watching that video. For our next activity, you are going to review the video of the patient encounter you completed earlier. We also have a transcript of the encounter for you to review while you watch the video.
>
> I am going to set up the laptop with your encounter video and give you the printed transcript. You can use the pen, pencil, or highlighter to make notes directly on the transcript as you reflect on the interaction.
>
> *Set up the laptop and open the participant's standardized patient encounter recording. Confirm that the correct participant video is loaded. Place the printed transcript, writing*

instruments, and written instruction sheet in front of the participant. Cue the video to the beginning of the encounter or to the first timestamp shown on the transcript, according to study procedures.

Here is a copy of the instructions for the activity. I will also review them with you now. Please watch the video of your patient encounter from beginning to end. You may pause the video at any time.

Point out the pause button.

Because we have limited time, we ask that you do not skip forward, skip backward, or replay sections of the video.

While watching your video, please pay attention to the words and phrases you used during the encounter. Apply what you learned in the video you just watched to identify things that you think went well and things that you might do differently in the future.

We encourage you to highlight specific words or phrases and make notes on the transcript about your language choices.

I will come back when 15 minutes have passed. If you finish watching the video before then, please continue reviewing, highlighting, or annotating the transcript. It is also completely okay if you do not make it all the way through the video. The purpose of this activity is reflection, so please do not feel rushed. Just review as much as you can during the time available.

There is not a natural place to take a break during this activity, so before we begin, would you like a moment to use the restroom? If you need something to drink, we also have water available.

Do you have any questions before we start?

Answer any procedural questions. Do not provide coaching or feedback on the participant's communication.

I am going to step into the hallway while you complete this activity. I will return when 15 minutes have passed.

Leave the Survey/Interview Room and start the 15-minute timer.

End of Activity

When the timer reaches 15 minutes, knock on the Survey/Interview Room door and re-enter.

We have reached the end of the 15 minutes, so we are going to move on to the next activity. Thank you for completing the reflection activity.

If needed, stop video playback. Collect or secure the study materials according to study procedures.
