# Supplementary material for: Promoting Stigma-Free Language in Type 2 Diabetes Care Among Primary Care Clinicians: Randomized Controlled Pilot Trial
Source: JMIR Diabetes. 2026 Aug 11;11:e95805. doi: 10.2196/95805 (PMC13460796; doi:10.2196/95805)
Supplement: Supplementary file 3 [file diabetes-v11-e95805-s003.pdf]

Multimedia Appendix 3. Theory of Planned Behavior Survey

Use of Language in Diabetes Care

The following survey was used to assess Theory of Planned Behavior domains related to clinicians' use of language in type 2 diabetes care. [1] The baseline survey assessed self-reported behavior, attitudes, subjective norms, perceived behavioral control, and intentions. The post-intervention survey repeated only the attitude and intention items.

The survey was administered electronically on a tablet. The language bank displayed at the top of each section remained visible while participants answered the corresponding items. The same item structure was repeated for each language bank.

In the instrument below, the phrase "words/statements" is used to refer to the active language bank displayed to the participant.

Participant Instructions

The following questions are about words and types of statements that are sometimes used in clinical interactions with people with type 2 diabetes.

Please answer each of the following questions by choosing the number that best describes your opinion. Some of the questions may appear to be similar, but they address somewhat different issues. Please read each question carefully.

Note: The term "appointment" in the questions is used to refer to:

- Verbal conversation with a patient during an appointment;
- Written instructions given to a patient at the end of an appointment (e.g., after visit summary)
- Written documentation of an appointment in the patient record (e.g., SOAP note)

Words
compliant<br>non-compliant<br>compliance<br>non-compliance

During the past 4 weeks, about how often have you used these words in your appointments with people with type 2 diabetes?

**All the time**						**Never**
○	○	○	○	○	○	○
1	2	3	4	5	6	7

For me, using these words in my appointments with people with type 2 diabetes is:

**Good**			**Neutral**			**Bad**
○	○	○	○	○	○	○
1	2	3	4	5	6	7

For me, using these words in my appointments with people with type 2 diabetes is:

**Useful**			**Neutral**			**Worthless**
○	○	○	○	○	○	○
1	2	3	4	5	6	7

Most health care providers like me use these words in their appointments with people with type 2 diabetes.

**Strongly Agree**						**Strongly Disagree**
○	○	○	○	○	○	○
1	2	3	4	5	6	7

My using or <u>not</u> using these words in my appointments with people with type 2 diabetes, is completely up to me.

**Strongly Agree**						**Strongly Disagree**
○	○	○	○	○	○	○
1	2	3	4	5	6	7

I intend to use these words in my appointments with people with type 2 diabetes, in the next 6 months.

**Strongly Agree**						**Strongly Disagree**
○	○	○	○	○	○	○
1	2	3	4	5	6	7

Words
Diabetic (as a noun)<br>(e.g., "Are you a diabetic?")

During the past 4 weeks, about how often have you used these words in your appointments with people with type 2 diabetes?

**All the time**						**Never**
○	○	○	○	○	○	○
1	2	3	4	5	6	7

For me, using these words in my appointments with people with type 2 diabetes is:

**Good**			**Neutral**			**Bad**
○	○	○	○	○	○	○
1	2	3	4	5	6	7

For me, using these words in my appointments with people with type 2 diabetes is:

**Useful**			**Neutral**			**Worthless**

○ ○ ○ ○ ○ ○ ○

1 2 3 4 5 6 7

Most health care providers like me use these words in their appointments with people with type 2 diabetes.

**Strongly Agree**						**Strongly Disagree**
○	○	○	○	○	○	○
1	2	3	4	5	6	7

My using or <u>not</u> using these words in my appointments with people with type 2 diabetes, is completely up to me.

**Strongly Agree**						**Strongly Disagree**
○	○	○	○	○	○	○
1	2	3	4	5	6	7

I intend to use these words in my appointments with people with type 2 diabetes, in the next 6 months.

**Strongly Agree**						**Strongly Disagree**
○	○	○	○	○	○	○
1	2	3	4	5	6	7

Words
Control (as a verb or an adjective)<br>(e.g., “controlled”, “uncontrolled”)<br>Control (as a noun)<br>(e.g., “glucose control”, “glycemic control”)

During the past 4 weeks, about how often have you used these words in your appointments with people with type 2 diabetes?

**All the time**						**Never**
○	○	○	○	○	○	○
1	2	3	4	5	6	7

For me, using these words in my appointments with people with type 2 diabetes is:

Good			Neutral			Bad
○	○	○	○	○	○	○
1	2	3	4	5	6	7

For me, using these words in my appointments with people with type 2 diabetes is:

Useful			Neutral			Worthless
○	○	○	○	○	○	○
1	2	3	4	5	6	7

Most health care providers like me use these words in their appointments with people with type 2 diabetes.

Strongly Agree						Strongly Disagree
○	○	○	○	○	○	○
1	2	3	4	5	6	7

My using or <u>not</u> using these words in my appointments with people with type 2 diabetes, is completely up to me.

Strongly Agree						Strongly Disagree
○	○	○	○	○	○	○
1	2	3	4	5	6	7

I intend to use these words in my appointments with people with type 2 diabetes, in the next 6 months.

Strongly Agree						Strongly Disagree
○	○	○	○	○	○	○
1	2	3	4	5	6	7

Words
poor, poorly<br>bad, badly<br>fail, failed

During the past 4 weeks, about how often have you used these words in your appointments with people with type 2 diabetes?

**All the time**						**Never**
○	○	○	○	○	○	○
1	2	3	4	5	6	7

For me, using these words in my appointments with people with type 2 diabetes is:

**Good**			**Neutral**			**Bad**
○	○	○	○	○	○	○
1	2	3	4	5	6	7

For me, using these words in my appointments with people with type 2 diabetes is:

**Useful**			**Neutral**			**Worthless**
○	○	○	○	○	○	○
1	2	3	4	5	6	7

Most health care providers like me use these words in their appointments with people with type 2 diabetes.

**Strongly Agree**						**Strongly Disagree**
○	○	○	○	○	○	○
1	2	3	4	5	6	7

My using or <u>not</u> using these words in my appointments with people with type 2 diabetes, is completely up to me.

**Strongly Agree**						**Strongly Disagree**
○	○	○	○	○	○	○
1	2	3	4	5	6	7

I intend to use these words in my appointments with people with type 2 diabetes, in the next 6 months.

**Strongly Agree**						**Strongly Disagree**
○	○	○	○	○	○	○
1	2	3	4	5	6	7

Statements focused on gain

(e.g., “More and more people are living long and healthy lives with diabetes. Let’s work together to make a plan that you can do in your daily life.”)

During the past 4 weeks, about how often have you used these words in your appointments with people with type 2 diabetes?

**All the time**						**Never**
○	○	○	○	○	○	○
1	2	3	4	5	6	7

For me, using these words in my appointments with people with type 2 diabetes is:

**Good**			**Neutral**			**Bad**
○	○	○	○	○	○	○
1	2	3	4	5	6	7

For me, using these words in my appointments with people with type 2 diabetes is:

**Useful**			**Neutral**			**Worthless**
○	○	○	○	○	○	○
1	2	3	4	5	6	7

Most health care providers like me use these words in their appointments with people with type 2 diabetes.

**Strongly Agree**						**Strongly Disagree**
○	○	○	○	○	○	○
1	2	3	4	5	6	7

My using or not using these words in my appointments with people with type 2 diabetes, is completely up to me.

**Strongly Agree**						**Strongly Disagree**
○	○	○	○	○	○	○
1	2	3	4	5	6	7

I intend to use these words in my appointments with people with type 2 diabetes, in the next 6 months.

**Strongly Agree**						**Strongly Disagree**

○	○	○	○	○	○	○
1	2	3	4	5	6	7

Statements focused on loss
(e.g., "You are going to end up on dialysis."; "You are going to end up blind."; "You are going to lose a foot.")

During the past 4 weeks, about how often have you used these words in your appointments with people with type 2 diabetes?

**All the time**						**Never**
○	○	○	○	○	○	○
1	2	3	4	5	6	7

For me, using these words in my appointments with people with type 2 diabetes is:

**Good**			**Neutral**			**Bad**
○	○	○	○	○	○	○
1	2	3	4	5	6	7

For me, using these words in my appointments with people with type 2 diabetes is:

**Useful**			**Neutral**			**Worthless**
○	○	○	○	○	○	○
1	2	3	4	5	6	7

Most health care providers like me use these words in their appointments with people with type 2 diabetes.

**Strongly Agree**						**Strongly Disagree**
○	○	○	○	○	○	○
1	2	3	4	5	6	7

My using or not using these words in my appointments with people with type 2 diabetes, is completely up to me.

**Strongly Agree**						**Strongly Disagree**
○	○	○	○	○	○	○
1	2	3	4	5	6	7

I intend to use these words in my appointments with people with type 2 diabetes, in the next 6 months.

**Strongly Agree**						**Strongly Disagree**
○	○	○	○	○	○	○
1	2	3	4	5	6	7

Scoring

Responses were recorded on 7-point scales. Domain scores were calculated by averaging the relevant items across all six language banks after recoding items so that higher scores consistently indicated movement away from stigmatizing language and toward person-first, strengths-based language.

The five non-recommended language banks were:

1. Authoritative language
    - Words
        - Compliant
          non-compliant
          compliance
          non-compliance

2. Non-person-centered language
    - Words
        - Diabetic (as a noun)
          (e.g., "Are you a diabetic?")

3. Morally implicative language
    - Words
        - Control (as a verb or an adjective)
          (e.g., "controlled", "uncontrolled")
          Control (as a noun)
          (e.g., "glucose control", "glycemic control")

4. Negative connotation language
    - Words
        - poor, poorly, bad, badly, fail, failed

5. Threatening language
    - Statements focused on loss
        - (e.g., "You are going to end up on dialysis."; "You are going to end up blind."; "You are going to lose a foot.")

The recommended language bank was:

1. Strengths-based language
    - Statements focused on gain
        - (e.g., "More and more people are living long and healthy lives with diabetes. Let's work together to make a plan that you can do in your daily life.")

For the self-reported behavior, attitude, subjective norm, and intention domains, items in the strengths-based language bank were reverse-scored before composite scores were calculated. This ensured that higher scores consistently reflected responses aligned with avoiding stigmatizing language and using person-first, strengths-based alternatives.

Perceived behavioral control was scored separately. Because stronger agreement with the behavioral control item indicated greater perceived control, all perceived behavioral control items were reverse-coded so that higher analytic scores represented greater perceived behavioral control over language use. The strengths-based language bank was not reverse-scored separately for the perceived behavioral control domain.

Domain Scores

- **Self-Reported Behavior**

  Item:
  "During the past 4 weeks, about how often have you used these words in your appointments with people with type 2 diabetes?"

  The self-reported behavior score was calculated as the mean of the six behavior items, one from each language bank, after reverse-coding the strengths-based language bank.

  Higher scores indicated less frequent use of stigmatizing language and more frequent use of strengths-based language.

- **Attitudes**

  Items:
  "For me, using these words in my appointments with people with type 2 diabetes is: Good–Bad."

  "For me, using these words in my appointments with people with type 2 diabetes is: Useful–Worthless."

  The attitude score was calculated as the mean of the 12 attitude items, two from each language bank, after reverse-coding the strengths-based language bank.

  Higher scores indicated less favorable attitudes toward stigmatizing language and more favorable attitudes toward strengths-based language.

- **Subjective Norms**

  Item:
  "Most health care providers like me use these words in their appointments with people with type 2 diabetes."

  The subjective norms score was calculated as the mean of the six subjective norm items, one from each language bank, after reverse-coding the strengths-based language bank.

  Higher scores indicated perceptions that other health care providers were less likely to use stigmatizing language and more likely to use strengths-based language.

- **Perceived Behavioral Control**

Item:
"My using or not using these words in my appointments with people with type 2 diabetes is completely up to me."

The perceived behavioral control score was calculated as the mean of the six perceived behavioral control items, one from each language bank, after reverse-coding the item direction so that higher scores represented greater perceived control.

Higher scores indicated greater perceived control over language use in clinical interactions with people with type 2 diabetes.

- **Intentions**

  Item:
  "I intend to use these words in my appointments with people with type 2 diabetes in the next 6 months."

  The intention score was calculated as the mean of the six intention items, one from each language bank, after reverse-coding the strengths-based language bank.

  Higher scores indicated lower intention to use stigmatizing language and greater intention to use strengths-based language.

Baseline and Post-Intervention Versions

At baseline, all five Theory of Planned Behavior domains were assessed: self-reported behavior, attitudes, subjective norms, perceived behavioral control, and intentions.

Immediately after the stigma-reduction training module or active control condition, only attitudes and intentions were reassessed. These domains were selected for post-intervention assessment because they were expected to be most responsive within the brief study visit window.

References

[1] Ajzen, I. The Theory of Planned Behavior. Organizational Behavior and Human Decision Processes. 1991 Dec;50(2):179–211. doi: 10.1016/0749-5978(91)90020-T
