# Supplementary material for: Promoting Stigma-Free Language in Type 2 Diabetes Care Among Primary Care Clinicians: Randomized Controlled Pilot Trial
Source: JMIR Diabetes. 2026 Aug 11;11:e95805. doi: 10.2196/95805 (PMC13460796; doi:10.2196/95805)
Supplement: Supplementary file 4 [file diabetes-v11-e95805-s004.pdf]

Multimedia Appendix 4. Acceptability Survey

The acceptability survey was administered after participants completed the Standardized Patient Encounter, Educational Video, and Guided Self-Reflection activity. Participants completed the survey independently on a tablet.

Before the survey, the Interviewer provided participants with a handout summarizing recommendations from the American Diabetes Association (ADA) and the Association of Diabetes Care & Education Specialists (ADCES) regarding language in diabetes care. [1]

Participants were instructed to answer the survey items based on those recommendations.

The survey assessed four acceptability domains:

1. Amount of information
2. Clarity of activities
3. Helpfulness of activities
4. Likelihood of recommending the activities to other health care providers

Interviewer Instructions and Participant Script

Staff-only directions are shown in *italicized brackets* and were not read aloud to participants. All other text was read aloud by the Interviewer.

> Next, we are going to go over some recommendations from a paper about language and diabetes outcomes.
>
> *Give participant the handout titled "Diabetes Language Recommendations."*
>
> I will briefly go through the recommendations with you.
>
> The American Diabetes Association and the Association of Diabetes Care & Education Specialists published recommendations for addressing the impact of language on diabetes outcomes. In the report, there are five key recommendations.
>
> They recommend:
>
> - Use language that is neutral, nonjudgmental, and based on facts, actions, or physiology/biology.
> - Use language free from stigma.
> - Use language that is strengths-based, respectful, and inclusive and that imparts hope.
> - Use language that fosters collaboration between people with diabetes and health care professionals.
> - Use language that is person-centered. For example, "person with diabetes" is preferred over "diabetic."
>
> Now, I am going to set up another survey for you. Please answer the following questions based on these recommendations.
>
> *Open the acceptability survey on the tablet and hand the tablet to the participant.*

Participant Handout

> **Diabetes Language Recommendations**
>
> The American Diabetes Association and the Association of Diabetes Care & Education Specialists published recommendations in 2017 addressing the impact of language on diabetes outcomes.
>
> The report includes five key recommendations:
>
> 1. Use language that is neutral, nonjudgmental, and based on facts, actions, or physiology/biology.
> 2. Use language free from stigma.

3. Use language that is strengths-based, respectful, and inclusive and that imparts hope.
4. Use language that fosters collaboration between people with diabetes and health care professionals.
5. Use language that is person-centered. For example, "person with diabetes" is preferred over "diabetic."

Questionnaire

The following questionnaire was originally programmed for self-administration on a tablet. In the programmed version, item labels such as "Amount," "Clarity," "Helpful," and "Recommend" were not visible to participants.

Participants could select an option to show the recommendations again while completing the survey.

Optional Display Text

Check here to show recommendations again.

The American Diabetes Association and the Association of Diabetes Care & Education Specialists published recommendations addressing the impact of language on diabetes outcomes. The report includes five key recommendations:

- Use language that is neutral, nonjudgmental, and based on facts, actions, or physiology/biology.
- Use language free from stigma.
- Use language that is strengths-based, respectful, and inclusive and that imparts hope.
- Use language that fosters collaboration between people with diabetes and health care professionals.
- Use language that is person-centered. For example, "person with diabetes" is preferred over "diabetic."

Please answer the following items based on these recommendations.

How would you describe the amount of information in today's activities in bringing awareness to these recommendations?

Too little information	Just the right amount of information	Too much information

○	○	○	○	○	○	○
1	2	3	4	5	6	7

How would you describe the clarity of today's activities in bringing awareness to these recommendations?

**Not clear at all**			**Somewhat clear**			**Extremely clear**
○	○	○	○	○	○	○
1	2	3	4	5	6	7

How helpful do you think today's activities might be for health care providers in bringing awareness to these recommendations?

**Not helpful at all**			**Somewhat helpful**			**Extremely helpful**
○	○	○	○	○	○	○
1	2	3	4	5	6	7

Would you recommend today's activities for health care providers in bringing awareness to these recommendations?

**No, I would strongly recommend against it**			**Not sure whether to recommend it or not**			**Yes, I would strongly recommend it**
○	○	○	○	○	○	○
1	2	3	4	5	6	7

Scoring and Interpretation

Each acceptability item was scored individually. No total acceptability score was calculated.

- **Amount of Information**

  The amount of information item was scored from 1 to 7:

  1 = Too little information

  4 = Just the right amount of information

  7 = Too much information

  For this item, higher scores do not necessarily indicate greater acceptability. A score near 4 indicates that the participant perceived the amount of information as appropriate.

- **Clarity**

  The clarity item was scored from 1 to 7:

  1 = Not clear at all

  4 = Somewhat clear

  7 = Extremely clear

  Higher scores indicate greater perceived clarity.

- **Helpfulness**

  The helpfulness item was scored from 1 to 7:

  1 = Not helpful at all

  4 = Somewhat helpful

  7 = Extremely helpful

  Higher scores indicate greater perceived helpfulness.

- **Likelihood of Recommendation**

  The recommendation item was scored from 1 to 7:

  1 = No, I would strongly recommend against it

  4 = Not sure whether to recommend it or not

  7 = Yes, I would strongly recommend it

  Higher scores indicate greater likelihood of recommending the activities to other health care providers.

References

[1] Dickinson JK, Guzman SJ, Maryniuk MD, O'Brian CA, Kadohiro JK, Jackson RA, D'Hondt N, Montgomery B, Close KL, Funnell MM. The Use of Language in Diabetes Care and Education. Diabetes Care. 2017 Dec;40(12):1790-1799. doi: 10.2337/dci17-0041
