# Supplementary material for: Promoting Stigma-Free Language in Type 2 Diabetes Care Among Primary Care Clinicians: Randomized Controlled Pilot Trial
Source: JMIR Diabetes. 2026 Aug 11;11:e95805. doi: 10.2196/95805 (PMC13460796; doi:10.2196/95805)
Supplement: Supplementary file 5 [file diabetes-v11-e95805-s005.pdf]

Multimedia Appendix 5. Semi-Structured Exit Interview

The semi-structured exit interview was conducted after participants completed the acceptability survey. The interview explored participants' prior exposure to diabetes language recommendations from the American Diabetes Association (ADA) and the Association of Diabetes Care & Education Specialists (ADCES),[1] perceived barriers and facilitators to accessing or implementing these recommendations, and feedback on the study activities.

The interviewer conducted the interview using a semi-structured guide displayed on the tablet.

The interview was audio-recorded.

The study activities referenced in the interview were:

- Standardized Patient Encounter
- Educational Video
- Guided Self-Reflection using the encounter recording
- Guided Self-Reflection using the encounter transcript

Interviewer Instructions and Participant Script

Staff-only directions are shown in *italicized brackets* and were not read aloud to participants. All other text was read aloud by the interviewer.

> We are going to move on to our last activity, which is an interview about your experience today.
>
> I will be recording this conversation on the tablet. When I first start the recording, I am going to say some information to label the recording, and then I will begin asking the interview questions.
>
> Are you ready for me to start the recording?
>
> *Open the audio recording software on the tablet. Begin the recording.*
>
> *Record the following audio label:*
>
> This is [Interviewer Name] recording for participant [Participant ID] on [Date] at [Time].
>
> The first set of questions is about the diabetes language recommendations. I am going to give you the handout again to remind you of them.
>
> *Give the participant the Diabetes Language Recommendations handout.*

Participant Handout

Diabetes Language Recommendations

The American Diabetes Association and the Association of Diabetes Care & Education Specialists published recommendations in 2017 addressing the impact of language on diabetes outcomes.

The report includes five key recommendations:

1. Use language that is neutral, nonjudgmental, and based on facts, actions, or physiology/biology.
2. Use language free from stigma.
3. Use language that is strengths-based, respectful, and inclusive and that imparts hope.
4. Use language that fosters collaboration between people with diabetes and health care professionals.
5. Use language that is person-centered. For example, "person with diabetes" is preferred over "diabetic."

Interview Guide

Staff-only interviewer notes and optional probes are shown in *italicized brackets*. Interviewers were permitted to use probes to clarify responses or elicit additional detail while maintaining a neutral, nonjudgmental tone.

Section 1. Prior Awareness of Diabetes Language Recommendations

I would like to first talk about your impression of these recommendations.

Question 1. Prior awareness

Have you heard of these diabetes language recommendations before today?

If yes, ask:

Can you help me understand your prior awareness of these recommendations?

Optional probes:

- How did you first hear about these recommendations?
- Where have you encountered these recommendations?

- Have these recommendations influenced your clinical communication in any way?

Question 2. Prior training

Have you participated in any type of training related to diabetes language, stigma-free communication, or improving communication with patients?

Optional probes:

- Have you participated in training on communicating with patients effectively?
- Have you participated in training focused specifically on diabetes communication?
- Have you participated in training focused on stigma, bias, or person-centered language?
- Can you tell me more about that training?

Section 2. Barriers and Facilitators

Question 3. Accessing and using recommendations

Can you tell me about any facilitators or barriers you perceive or experience in accessing, understanding, or using recommendations like these?

Optional probes about barriers:

- Have you experienced any barriers to accessing information such as these recommendations?
- Are there barriers to using these recommendations in clinical practice?
- Are there workflow, time, documentation, training, or institutional factors that make these recommendations easier or harder to use?

Optional probes about facilitators:

- What would make it easier to access information such as these recommendations?
- What would make it easier to use these recommendations in clinical practice?
- Are there specific resources, tools, reminders, or training formats that would be helpful?

Section 3. Feedback on Study Activities

For the next questions, I would like you to think about the activities you completed today.

These activities included:

- the standardized patient encounter;
- the educational video;
- reviewing your standardized patient encounter recording; and
- reviewing or annotating your standardized patient encounter transcript.

For each question in this section, probe as appropriate for each activity listed above.

Question 4. Overall experience

What was this experience like for you?

Optional probes:

- What stood out to you most?
- Was any part of the experience particularly useful?
- Was any part of the experience uncomfortable, unclear, or difficult?
- How did the standardized patient encounter feel?
- How did it feel to review your own encounter recording?
- How did it feel to review or annotate the transcript?

Question 5. Helpfulness for awareness

From your perspective, were the activities you participated in today helpful in bringing awareness to the impact of language in diabetes care?

Optional probes:

- Which activity was most helpful for bringing awareness to language use?
- Was any activity less helpful?
- Did any activity change the way you think about language used in diabetes care?
- Did the activities help you identify anything about your own communication?

Question 6. Helpfulness for other health care providers

In your opinion, might these activities be helpful for other health care providers in gaining awareness or understanding of these recommendations?

Optional probes:

- Which types of health care providers might benefit most?
- Would this be useful for clinicians in training, practicing clinicians, or both?
- Would this be useful in primary care settings?
- Are there any groups for whom this type of activity might be less useful?

Question 7. Suggested changes

Is there anything you would change about these activities to make them more useful for other health care providers?

Optional probes:

- Would you change the standardized patient encounter?
- Would you change the educational video?
- Would you change the self-reflection activity?
- Would you change the transcript review or annotation process?
- Would you change the length, format, or setting of the activities?
- Would you add anything?

Closing

Those are all of the interview questions I have. Is there anything else you would like to share about your experience today or about language use in diabetes care?

After the participant responds, stop the audio recording.

Thank you for sharing your thoughts and for participating in the study.

Confirm that the audio file has been saved according to study procedures. Advance to the next section of the protocol.

[1] Dickinson JK, Guzman SJ, Maryniuk MD, O'Brian CA, Kadohiro JK, Jackson RA, D'Hondt N, Montgomery B, Close KL, Funnell MM. The Use of Language in Diabetes Care and Education. Diabetes Care. 2017 Dec;40(12):1790-1799. doi: 10.2337/dci17-0041
