# Supplementary material for: Promoting Stigma-Free Language in Type 2 Diabetes Care Among Primary Care Clinicians: Randomized Controlled Pilot Trial
Source: JMIR Diabetes. 2026 Aug 11;11:e95805. doi: 10.2196/95805 (PMC13460796; doi:10.2196/95805)
Supplement: Supplementary file 6 [file diabetes-v11-e95805-s006.pdf]

Multimedia Appendix 6. Sensitivity analyses by analytic approach

**Outcome**	**Analysis approach**	**n, SRM/AC**	**F statistic**	**p-value**	**Interpretation**
Attitudes	As-treated	12/9	14.27	.001	Significant condition × time interaction
Attitudes	As-randomized	11/10	10.42	.004	Same conclusion
Attitudes	Per-protocol	11/9	13.02	.002	Same conclusion
Intentions	As-treated	12/9	2.33	.143	Not statistically significant
Intentions	As-randomized	11/10	4.68	.044	Significant in this analysis only
Intentions	Per-protocol	11/9	3.57	.075	Not statistically significant

SRM = stigma-reduction module; AC = active control.
As-treated analyses classified participants according to the condition received. As-randomized analyses classified participants according to original randomized allocation. Per-protocol analyses excluded the participant affected by the programming error.
The n column presents the number of participants in the SRM and AC conditions for each analytic approach.
F statistics and p-values are for the condition × time interaction from 2 × 2 repeated-measures ANOVA models.
Because the statistical significance of the intention outcome differed across analytic approaches, intention findings were interpreted cautiously as exploratory.
